# Managing a locally advanced malignant thymoma complicated by nephrotic syndrome: a case report

**DOI:** 10.1186/1752-1947-2-89

**Published:** 2008-03-19

**Authors:** Daren CY Teoh, Ahmed El-Modir

**Affiliations:** 1 Cancer Centre, Queen Elizabeth Hospital, Birmingham, B15 2TH, UK

## Abstract

**Introduction:**

The management of locally advanced inoperable malignant thymoma is difficult as there are no large randomized clinical trial data to guide treatment. However various case series have shown that malignant thymoma is often a chemosensitive disease. Cisplatin-based chemotherapy has been the gold-standard in the management of these patients. However when thymic cancers are complicated by paraneoplastic syndromes that damage kidney and neurological function, cisplatin use is often contraindicated.

**Case presentation:**

We report a case of a 37 year old man with locally advanced malignant thymoma complicated by significant nephrotic syndrome and renal impairment. He responded to a novel combination of carboplatin, epirubicin and cyclophosphamide chemotherapy used as first line therapy.

**Conclusion:**

The treatment with chemotherapy of locally advanced malignant thymoma complicated by nephrotic syndrome and renal impairment is difficult due to the increase of toxicity. In this case, a novel chemotherapy combination with lesser toxicity was used successfully. In addition this chemotherapy combination did not impede the later use of conventional cisplatin-based chemotherapy. Therefore we suggest a course of carboplatin-based chemotherapy for locally advanced malignant thymoma in patients who are unsuitable for cisplatin.

## Introduction

The management of locally advanced inoperable malignant thymoma is difficult. There is no large randomized clinical trial data to guide treatment. However various case series have shown that malignant thymoma is often a chemosensitive disease. Chemotherapy can be used neoadjuvantly to downstage disease rendering inoperable disease operable or as palliative treatment to extend life and improve its quality. In 1993, Berruti et al used doxorubicin, cisplatin, vincristine and cyclophosphamide neoadjuvantly[1]. Hosokawa et al used a combination of cisplatin, vincristine, doxorubicin and etoposide to render an inoperable invasive thymoma operable[2]. Many case series have reported >50% response rates with cisplatin-based chemotherapy and this has now become the standard of care for inoperable or metastatic malignant thymomas[3].

However not all patients are suitable for cisplatin-based chemotherapy. In particular, patients with renal or neurological impairment are not suited to the renal and neuro-toxicities of cisplatin. This is particularly pertinent as thymomas may present with associated renal impairment and up to 35% have associated myasthenia gravis[4].

In this case report, we describe a challenging case of recurrent locally advanced malignant thymoma complicated by renal impairment and nephrotic syndrome. In view of this, the less nephrotoxic platinum – carboplatin was used. We describe our experience with carboplatin in combination with epirubicin, cyclophosphamide chemotherapy which to our knowledge is a yet untested combination in the primary treatment of locally advanced inoperable thymoma.

## Case presentation

We report a case of a 37 year old man of Indian ethnicity who had a thymectomy 16 years ago, in Oct 1989. He had presented with myasthenia gravis. Perioperatively the tumour was found to be adherent to the pericardium, left phrenic nerve, anterior chest wall and the left lung. A complete resection was reported and he did not receive chemotherapy or adjuvant radiotherapy at that time.

**Figure 1:**
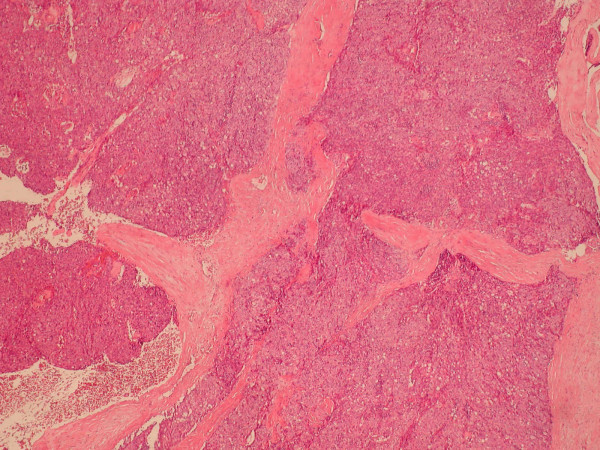
Haematoxylin and eosin stains from biopsy of the thymic tumour.

Apart from mild myasthenic symptoms managed with pyridostigmine, he had been well until early 2005 when he developed new left sided chest pain. CT scan of the thorax from March 2005 showed extensive involvement of the pleura on the left side with penetration of the diaphragm and invasion of the spleen (Masouka Stage IVA). The tumour was also wrapping around the aortic arch. He underwent an exploratory left thoracotomy in May 2005, but unfortunately surgical debulking was not possible and pleural biopsies were taken. The histology report was of a recurrent well differentiated thymic carcinoma (Fig. 1).

**Figure 2:**
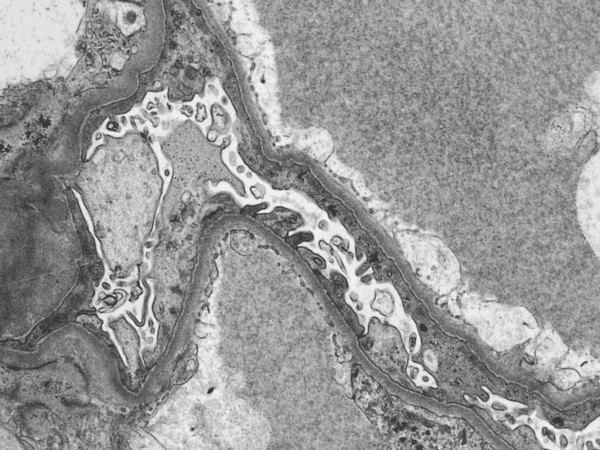
Electron microscopy demonstrating minimal change nephropathy.

Postoperatively he developed acute renal impairment and peripheral oedema. A renal biopsy in May 2005 confirmed minimal change nephropathy (Fig. 2). His renal impairment may have also been made worse by a degree of acute tubular necrosis post-operatively. At this stage his serum creatinine was 172 umol/L with a creatine clearance of 42 ml/min and he had significant proteinuria of 24 g/L with a serum albumin of 15 g/L. His WHO performance status was 1. He was therefore started on prednisolone 40 mg daily as initial treatment for his nephrotic syndrome and was subsequently referred for chemotherapy.

Due to his renal dysfunction and proteinuria, he was initially commenced in June 2005 on epirubicin and cyclophosphamide (EC) chemotherapy at reduced doses of 37.5 mg/m^2 ^and 300 mg/m^2 ^respectively. Cycles were repeated every 3 weeks. 2^nd ^and subsequent cycles were given at full doses of 70 mg/m^2 ^and 600 mg/m^2^.

After 2 cycles of chemotherapy and 2 months treatment with prednisolone 40 mg daily, his nephropathy improved to a serum creatinine of 63 umol/L, serum albumin of 25 g/L and his proteinuria to under 6 g/L. This allowed for the addition of a platinum chemotherapy agent and for his steroid dose to be gradually tapered by approximately 5 mg every 2 weeks. In view of his recent nephropathy and ongoing proteinuria – carboplatin was chosen over cisplatin. The initial carboplatin dose was AUC 3.5 (calculated using the Calvert formula) and this was increased to AUC 4 and subsequently to AUC 5 in the following 4 cycles of carboplatin combination chemotherapy. He tolerated treatment extremely well and his renal function remained in the normal range throughout these 4 cycles. On completion of the 6 cycles of chemotherapy his serum creatinine was 81 umol/L, serum albumin 39 g/L and proteinuria 0.34 g/L.

He had one admission to hospital due to epigastric pain and vomiting prior to his 2^nd ^cycle of chemotherapy. This may have been brought on by the combination of chemotherapy and high dose steroids. This promptly settled on high dose omeprazole. He also had grade 1 joint aches and a bursitis of the left elbow which settled on oral antibiotics.

**Figure 3:**
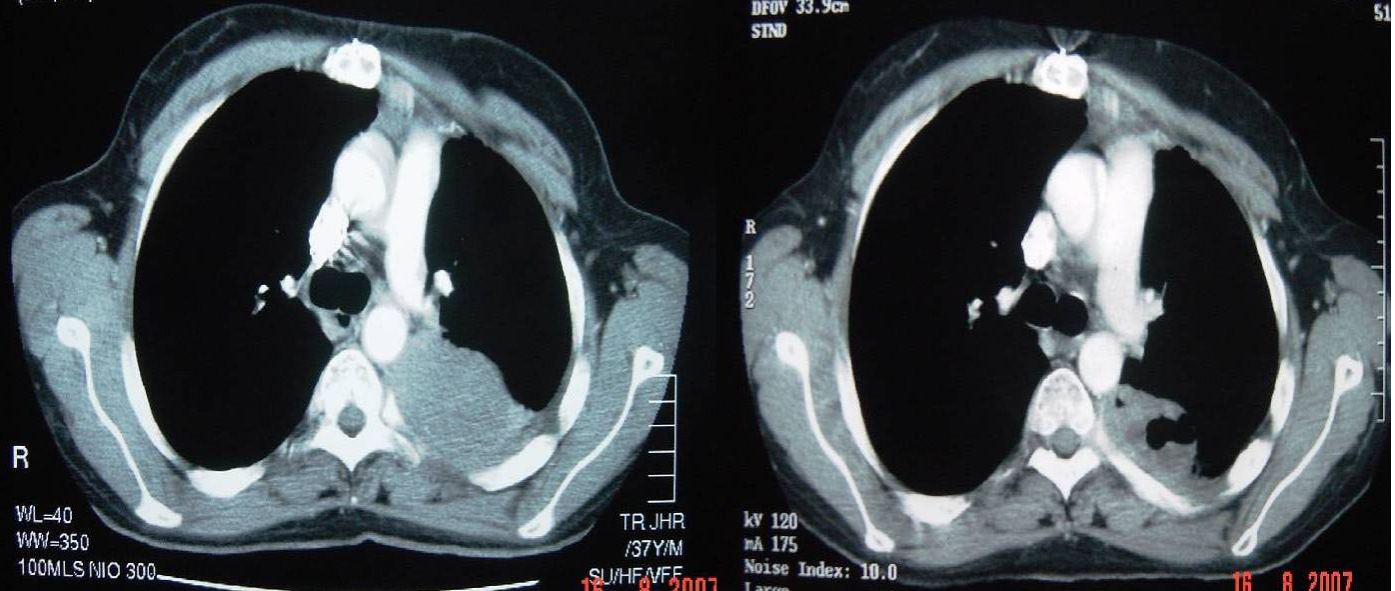
Pre- and post-treatment CT scans at the level of the carina.

A CT scan in October 2005, 2 weeks after his 6^th ^and last cycle of chemotherapy, showed a good partial response in comparison with scans taken pre-chemotherapy (Fig. 3). He remained symptom-free and without disease progression until February 2006. Time to progression was 4 months. He went on to receive 2^nd ^line chemotherapy in the form of etoposide, ifosfamide and cisplatin chemotherapy for a further 6 cycles. His minimal change nephropathy remained in remission at this time allowing for the safe introduction of cisplatin. He attained a good partial response again from this regime but subsequently progressed again in April 2007. He remains alive and independent at the time of submission of this report.

## Conclusion

Thymic tumours may rarely be complicated by nephrotic syndrome at the time of initial diagnosis, on recurrence or even in remission[5]. The cellular pathology linking these two conditions have yet to be fully explained, although T-cell dysfunction has been suggested[5]. The vast majority of nephrotic syndrome cases associated with thymic tumours are due to minimal change nephropathy – and the mainstay of treatment is with high dose steroids and treatment of the primary tumour.

However chemotherapy treatment of thymic tumours complicated by nephrotic syndrome requires special consideration. Nephrotic syndrome confers an increased risk of infection due to the lost of immunoglobulins. Thus the risk of neutropaenic sepsis with chemotherapy is greater. In this case, we managed the risk of neutropaenic infection by gradually titrating the dose upwards as the proteinuria improved and treating any signs of infection without delay.

Classically cisplatin-based chemotherapy has been used in the treatment of locally advanced or metastatic malignant thymoma. Hanna et al at Indiana University did use high-dose carboplatin (700 mg/m2) with etoposide in patients with recurrent thymoma but in association with peripheral blood stem cell rescue[6]. Jan et al reported a case of recurrent thymoma in which carboplatin and paclitaxel chemotherapy was used in the 2nd line setting which resulted in an improvement of clinical symptoms and reduction in the tumour mass[7]. The authors suggested further investigation on the use of carboplatin in the 1^st ^line setting.

To our knowledge carboplatin has not been used in the 1^st ^line treatment of locally advanced or recurrent thymoma. The advantage of carboplatin over cisplatin is its lesser nephrotoxicity and neurotoxicity. These characteristics are particularly useful as thymomas are frequently associated with paraneoplastic syndromes which often affect renal and/or neurological function as demonstrated in this case[4]. This case also demonstrates that carboplatin use upfront did not impede the subsequent responsiveness of the tumour to cisplatin-based chemotherapy.

Doxorubicin has also been the anthracycline of choice in most other reported cases of chemotherapy for malignant thymomas. Macchiarini et al used epirubicin in combination with cisplatin and etoposide but in the neoadjuvant setting[8]. As epirubicin may be less cardio-toxic compared with doxorubicin, it may be a better choice especially if thoracic radiotherapy will be or had been used previously. In addition, cyclophosphamide was used initially rather than ifosfamide. Ifosfamide was not used initially as his low serum albumin would have increased the risk of ifosfamide-induced encephalopathy. Furthermore steroid-resistant nephrotic syndrome has been shown to respond to cyclophosphamide and therefore it may have had a dual benefit of treating both nephropathy and tumour in this case[9].

Locally advanced inoperable malignant thymoma complicated by nephrotic syndrome presents a challenge to conventional treatment. Although this is usually a chemosensitive tumour, the toxicity of traditional agents such as cisplatin may prohibit their use. Can carboplatin therefore be considered an equivalent alternative to cisplatin in the chemotherapy treatment of thymic cancers? In the treatment of ovarian or small cell lung cancer, carboplatin has been shown to be broadly equivalent to cisplatin. Conversely in the treatment of germ cell tumours it has been shown to be clearly inferior[10]. The above case demonstrates that carboplatin-based chemotherapy has activity against locally advanced inoperable thymic cancers in the 1^st ^line setting. In patients who have renal or neurological impairment, we suggest that carboplatin would be the better alternative. However in those who could tolerate cisplatin, a head-to-head clinical trial would be needed, but may be difficult due to the relative rarity of thymic cancers. In the absence of strong clinical data, we suggest a course of carboplatin-based chemotherapy for locally advanced malignant thymoma in patients who are unsuitable for cisplatin.

## Competing interests

The author(s) declare that they have no competing interests.

## Authors' contributions

DT collected the data and wrote the report whilst AEM contributed to the script. Both were involved in the care of the patient reported and both have read and approved the final script.

## Consent

Written informed consent was obtained from the patient for publication of this case report and accompanying images. A copy of the written consent is available for review by the Editor-in-Chief of this journal.
